# Improving laboratory animal genetic reporting: LAG-R guidelines

**DOI:** 10.1038/s41467-024-49439-y

**Published:** 2024-07-02

**Authors:** Lydia Teboul, James Amos-Landgraf, Fernando J. Benavides, Marie-Christine Birling, Steve D. M. Brown, Elizabeth Bryda, Rosie Bunton-Stasyshyn, Hsian-Jean Chin, Martina Crispo, Fabien Delerue, Michael Dobbie, Craig L. Franklin, Ernst-Martin Fuchtbauer, Xiang Gao, Christelle Golzio, Rebecca Haffner, Yann Hérault, Martin Hrabe de Angelis, Kevin C. Kent Lloyd, Terry R. Magnuson, Lluis Montoliu, Stephen A. Murray, Ki-Hoan Nam, Lauryl M. J. Nutter, Eric Pailhoux, Fernando Pardo Manuel de Villena, Kevin Peterson, Laura Reinholdt, Radislav Sedlacek, Je Kyung Seong, Toshihiko Shiroishi, Cynthia Smith, Toru Takeo, Louise Tinsley, Jean-Luc Vilotte, Søren Warming, Sara Wells, C. Bruce Whitelaw, Atsushi Yoshiki, Asian Mouse Mutagenesis Resource Association, Atsushi Yoshiki, Chi-Kuang Wang, CELPHEDIA infrastructure, Jacqueline Marvel, Ana Zarubica, INFRAFRONTIER consortium, Sara Wells, International Mammalian Genome Society, International Mouse Phenotyping Consortium, Jason Heaney, Sara Wells, International Society for Transgenic Technologies, Mutant Mouse Resource and Research Centers, Ian F. Korf, Cathleen (Cat) Lutz, Phenomics Australia, Andrew J. Kueh, Paul Q. Thomas, Ruth M. Arkell, Graham J. Mann, RRRC- Rat Resource and Research Center, Guillaume Pavlovic

**Affiliations:** 1 The Mary Lyon Centre at MRC Harwell, Harwell Campus, Didcot, OX11 0RD Oxon UK; 2 University of Missouri School of Medicine, Columbia, MO USA; 3 University of Missouri College of Veterinary Medicine, Columbia, MO USA; 4 Rat Resource and Research Center, University of Missouri, Columbia, MO USA; 5 Department of Epigenetics and Molecular Carcinogenesis, The University of Texas MD Anderson Cancer Center, Houston, TX USA; 6 PHENOMIN-Institut Clinique de la Souris, CELPHEDIA, CNRS, INSERM, Université de Strasbourg, Illkirch-Grafenstaden, 67404 Strasbourg, France; 7 Visiting Scientist, Institut Clinique de la Souris, Université de Strasbourg, Illkirch-Grafenstaden, 67404 Strasbourg, France; 8 Rat Resource and Research Center, University of Missouri, Columbia, MO 65201 USA; 9 National Laboratory Animal Center (NLAC), NARLabs, Taipei, Taiwan; 10 Laboratory Animal Biotechnology Unit, Institut Pasteur de Montevideo, Mataojo 2020, CP 1400 Montevideo, Uruguay; 11 Department of Genetics, The University of Texas MD Anderson Cancer Center, Houston, TX USA; 12 Phenomics Australia, Australian National University, 131 Garran Road, Canberra, ACT 2601 Australia; 13 University of Missouri Mutant Mouse Resource and Research Center (MU MMRRC), University of Missouri, Columbia, MO 65201 USA; 14 Department of Molecular Biology, University of Aarhus, Aarhus C, 8000 Denmark; 15 National Resource Center of Mutant Mice (NRCMM), Nanjing Biomedical Research Institute, Nanjing University, Nanjing, China; 16 Université de Strasbourg, CNRS, Inserm, IGBMC UMR 7104- UMR-S 1258, F-67400 Illkirch, France; 17 Department Veterinary Resources, Weizmann Institute of Science, Rehovot, Israel; 18 Institute of Experimental Genetics, Helmholtz Zentrum München, German Research Center for Environmental Health (GmbH), Ingolstaedter Landstraße 1, 85764 Neuherberg, Germany; 19 Chair of Experimental Genetics, TUM School of Life Sciences, Technische Universität München, Alte Akademie 8, 85354 Freising, Germany; 20 German Center for Diabetes Research (DZD), Ingolstaedter Landstraße 1, 85764 Neuherberg, Germany; 21 Mouse Biology Program, University of California Davis, Davis, CA USA; 22 Department of Genetics, and Lineberger Comprehensive Cancer Center, The University of North Carolina at Chapel Hill, Chapel Hill, NC 27599-7264 USA; 23 Department of Molecular and Cellular Biology, National Centre for Biotechnology (CNB-CSIC), 28049 Madrid, Spain; 24 Centre for Biomedical Network Research on Rare Diseases (CIBERER-ISCIII), 28029 Madrid, Spain; 25 The Jackson Laboratory, Bar Harbor, ME USA; 26 Laboratory Animal Resource Center, Korea Research Institute of Bioscience and Biotechnology (KRIBB), Daejeon, Korea; 27 Genetics and Genome Biology, The Hospital for Sick Children and The Centre for Phenogenomics, Toronto, ON M5T 3H7 Canada; 28 Université Paris-Saclay, UVSQ, INRAE, BREED, 78350 Jouy-en-Josas, France; 29 Department of Genetics, University of North Carolina, Chapel Hill, NC 27599 USA; 30 Lineberger Comprehensive Cancer Center, University of North Carolina, Chapel Hill, NC 27599 USA; 31 Czech Centre for Phenogenomics, Institute of Molecular Genetics of the Czech Academy of Sciences, Prague, Czech Republic; 32 Laboratory of Developmental Biology and Genomics, BK21 PLUS Program for Creative Veterinary Science Research, Research Institute for Veterinary Science, College of Veterinary Medicine, Seoul National University, and Korea Mouse Phenotyping Center, Seoul, 08826 Republic of Korea; 33 RIKEN BioResource Research Center (BRC), Tsukuba, Japan; 34 Mouse Genome Informatics (MGI), Jackson Laboratory, Bar Harbor, ME USA; 35 Center for Animal Resources and Development (CARD), Institute of Resource Development and Analysis, Kumamoto University, Kumamoto, Japan; 36 Université Paris-Saclay, INRAE, AgroParisTech, GABI, Jouy-en-Josas, France; 37 Genentech, Inc., a member of the Roche group, South San Francisco, CA USA; 38 Francis Crick Institute, London, NW1 1AT UK; 39 The Roslin Institute and Royal (Dick) School of Veterinary Studies, University of Edinburgh, Easter Bush, EH25 9RG UK; 40 Experimental Animal Division, RIKEN BioResource Research Center, Tsukuba, Ibaraki 305-0074 Japan; 41 CIRI - Centre International de Recherche en Infectiologie, Université Claude Bernard Lyon Inserm, U1111, CNRS, UMR5308, ENS Lyon, Lyon, France; 42 Centre d’Immunophénomique (CIPHE), Aix Marseille Université, INSERM, CNRS, CELPHEDIA, PHENOMIN, Marseille, France; 43 Department of Molecular and Human Genetics, Baylor College of Medicine, Houston, TX USA; 44 University of California Davis, Department of Molecular and Cellular Biology; UC Davis Genome Center, California, USA; 45 Genome Engineering and Cancer Modelling Program, Olivia Newton-John Cancer Research Institute, Melbourne, VIC Australia; 46 School of Cancer Medicine, La Trobe University, Melbourne, VIC Australia; 47 School of Biomedicine, University of Adelaide, North Terrace, Adelaide, SA 5005 Australia; 48 John Curtin School of Medical Research, Australian National University, 131 Garran Road, Canberra, ACT 2601 Australia

**Keywords:** Eukaryote, Animal breeding, CRISPR-Cas9 genome editing, Sequencing

## Abstract

The biomedical research community addresses reproducibility challenges in animal studies through standardized nomenclature, improved experimental design, transparent reporting, data sharing, and centralized repositories. The ARRIVE guidelines outline documentation standards for laboratory animals in experiments, but genetic information is often incomplete. To remedy this, we propose the Laboratory Animal Genetic Reporting (LAG-R) framework. LAG-R aims to document animals’ genetic makeup in scientific publications, providing essential details for replication and appropriate model use. While verifying complete genetic compositions may be impractical, better reporting and validation efforts enhance reliability of research. LAG-R standardization will bolster reproducibility, peer review, and overall scientific rigor.

## Abstract (web summary)

Reproducibility is key to all fields of research, and in the case of animal research, this can be hampered by inconsistent reporting of animal genetics. Here the authors propose a reporting framework and guidelines designed to discuss the validation and standardise the reporting of genetic backgrounds and genetic alterations in animal models.

## Introduction

The biomedical research community has recognized many of the factors leading to irreproducibility of animal research^1,2^ and has begun to implement solutions to address this challenge: these cover many aspects, including further investment in standardized nomenclature^3,4^, improved experimental design (PREPARE^5^), reporting of animal research (ARRIVE, Animal Research: Reporting of In Vivo Experiments^6^) and open data sharing^7^. In this context, recommendations for a common standard of documentation for animal experiments^6^ serve a particularly important purpose.

The ARRIVE guidelines list the key aspects for documenting laboratory animals used in experiments: information on individuals, metadata, experimental procedures and study design^6^. The guidelines also recommend core documentation on animal model characteristics, such as genetic modification status, genotype, and manipulated gene(s), as well as genetic methods and technologies used to generate and validate the animals^8^. However, in addition to environmental factors (sanitary status, diet, etc.), the genotype–phenotype relationship can be markedly influenced by the genetic background of experimental animals^9,10^ and the reproducibility of the genotype–phenotype relationship is significantly impacted by breeding paradigms, source colony, and genetic drift^11^. Even differences in genetics, too often perceived as subtle, for example, the differences between C57BL/6 substrains in mice, can have a significant impact^12^. Therefore, a need remains for a more comprehensive description of the genetics of research animals to enable data interpretation and reproducibility. Such information is also needed to allow other scientists to acquire, maintain, and use experimental models for investigations building on published data. Here, we propose a framework of reporting guidelines, complementary to the ARRIVE guidelines, to support the documentation in scientific publications of the genetics of animals used in research. To be clear, this framework does not aim to impose standardization of animal genetics^13^, but, rather, to improve the documentation associated with animals used for scientific research. It is intended to be applicable to all animals used for research.

Our proposed framework applies to the full range of animal species used in life-science research and, in the case of genetically engineered animals, to those modified by either classical or current methods of genome engineering. Here, we discuss how this reporting framework is designed to document the genetic background and validation (defined here as the act of verifying) of animal models and to link this information to infrastructures that support the community in sharing data and materials. We also examine the fundamentals of genetic validation for animal models and we present the role of supporting frameworks for reporting animal genetics. We call these recommendations the Laboratory Animal Genetic Reporting (LAG-R) guidelines. By standardizing information, the LAG-R guidelines will improve the sharing and replicability of models across research teams and will guide peer reviewers in their assessment of the essential genetic information for animal models provided in manuscripts.

## Reporting genetic backgrounds and genetic alterations

The limitations of current standards for comprehensive reporting of the genetics of animals in research publications have been the subject of many discussions, both informal and structured, among relevant research societies and consortia in recent years. The lack of standardization in reporting currently results in a deterioration of information regarding research animal models, particularly as they transition between different laboratories. This has many negative consequences, particularly if the animal model was not fully described in the initial publication and subsequent breeding records are partial or absent. This can prevent re-analysis of data, as fundamental and basic variables on research materials are not documented. It can also lead to misinterpretation of published studies. In addition, imprecise definition of genetic background may lead to the use of experimental animals with different genetics and phenotypes in subsequent studies, which is a known contributing factor in true or perceived irreproducibility of research^14^. This wastes significant research resources^15^, including those used to reconstitute missing information on both genetic background and genetic alterations^2^, as well as those used to re-establish genotyping assays^16^. The worst possible consequence can be the waste of experimental animals. By contrast, appropriate documentation and reporting will contribute to reduction and refinement practices in keeping with the 3Rs^17^ and will result in better management of animal use^6^.

The development and implementation of documentation and reporting standards of laboratory animal genetics present an opportunity to improve research reproducibility. Here, we propose two sets of features to be documented (Table 1): the first is applicable to all laboratory animals; the second, with additional criteria, is to document the genetic alterations of engineered and other genetic models.

**Table 1:** Minimal information needed for correct identification of species, lineages, and genetic alterations

Criteria to report on for all research animals^a^		
Genetic background description, including Strain/breed/stock type	1	**Official name of species, strain, and sub-strain, as applicable, of the animal. Alternatively, for farm animals, indicate breed** https://www.ncbi.nlm.nih.gov/Taxonomy/Browser/wwwtax.cgi. **Describe type of breeding for strain/stock/breed using species standard wording**. **For example, in rodents: outbred, inbred, hybrid, congenic**^b^**, documented mixed or non-documented genetic background. For example, in zebrafish: specify which wildtype background (AB, TU, TL); if mixed background, provide a clear explanation of the breeding scheme and estimation of the percentage of each background**.
Breeding scheme and stability program (only relevant for rodents)^c^	2	**Specify breeding schemes used to maintain stock and generate experimental animals. Include the genotype of the parents when possible. This is particularly important to trace the origin of sex chromosomes in congenic strains**^b^. *Specify breeding strategy to maintain genetic quality of the colony; indicate known family tree*.
Source of animals	3	**Name origin of strain(s). Name supplier or repository or other origin of animals used in the experiment**.
International nomenclature	4	**Name strain according to internationally agreed standard when available**^c^. *Use research resource identifier (RRID) when applicable*.
*Strain or stock identifier*	5	*Show unique identifier of strain or stock used by the supplier or the repository*.
*Genetic background validation*	6	*Indicate if, when (at what breeding generation) and how the genetic background was verified (i.e., sequencing, SNP (single-nucleotide polymorphism) panel, STR panel, genetic testing chip panel)*.

Additional criteria to report on for animals with genetic alteration^a^		
Name of mutant allele	7	**Detail the shorthand used in article, and official nomenclature**^d^. *Use unique identifier (e.g. MGI ID) when applicable*.
Allele type	8	**Specify the method of model creation: naturally occurring allele/gene targeting/genome editing/additive transgenesis/chemical or physical mutagenesis/viral insertion/site-specific recombination/transposition**.
*Intended and observed consequence of mutagenesis*	9	*Detail whether allele is a frameshift, deletion, coding or non-coding variant, overexpression, conditional allele, humanization, reporter, structural variation. Detail new gene product if known*.
Model summary description	10	**Provide a short summary of genetic modification and background used for establishing genetic alteration**.
DNA sequence	11	**Provide access to the sequence of the genetic modification: targeting vector, donor template or vector for transgenesis. If employed in the mutagenesis process, provide the sequence of donor (e.g., targeting vector, oligodeoxynucleotide, transgene or template sequence used for mutagenesis; DNA or prime editing guide)**^e^. *Annotate genomic sequences with corresponding genome assembly version and coordinates. Use universal format; i.e.,.fasta or.gb. Annotate features*.
*Allele schematic*	12	*Consider presenting a map of the genetic modification*.
Material availability/source of materials	13	**Describe how to access available materials (plasmids, mutant cells, animals and/or germplasm)**. *RRID and/or repository identifier*.
Obvious phenotype and welfare concern	14	**Specify salient phenotypes, such as issues with viability and/or fertility, or immunodeficiency. Describe the severity of the associated phenotype. If necessary, include any requirement to mitigate welfare concerns. Include publication, archive or database reference if available**. *For mice, consider SHIRPA (SmithKline Beecham/Harwell/Imperial College/Royal London Hospital/phenotype assessment) description*^60^.
Initial reference	15	**Detail whether report is the initial description of mutant and/or mention initial publication of materials**.
Genotyping assay	16	**Describe assay and sequence of primers used for genotyping of established colony**.
Enzyme and other reagents used for genome engineering	17	**Describe enzymes (nuclease, recombinase) if used to generate mutation including number and sequence of guide(s) for ribonucleoproteins if relevant. Detail reagents**^f^.
Validation of allele sequence	18	*If done, describe how the region of interest was validated*^g^.
Validation of allele structure	19	*If done, describe the precise method used for validation of chromosomal or allele structure, and the outcome*^g^.
Validation to exclude additional integration of mobilized sequence	20	*If done, describe the method and outcome of analyzing the material for additional integrations of donor templates*^e^ *or reintegration of deleted segments*^g^.
Evaluation of potential off-target activity	21	*Genome editing off-target is defined as a genomic position and/or nucleic acid sequence distinct from the target. If done, describe the method, selection criteria and outcome of off-target analysis*.

^a^Essential criteria are indicated in bold; recommended criteria are indicated in italic. The information itself, or a reference to a source, should be detailed.

^b^Congenic strains are examples of the importance of correct breeding scheme, as their genetic composition varies according to the parental origin of the sex chromosomes and the mitochondrial genome. In addition, the identity of the region around a transmitted allele remains that of the original strain.

^c^Definitions and guidelines for nomenclature of mouse and rat strains are described at https://www.informatics.jax.org/mgihome/nomen/strains.shtml.

^d^Guidelines for Nomenclature of Genes, Genetic Markers, Alleles, and Mutations in Mouse and Rat are described at https://www.informatics.jax.org/mgihome/nomen/gene.shtml.

^e^Note that donor sequence can differ from mutagenesis outcome.

^f^Some recommendations for genome-editing formulations are reported in ref. ^61^.

^g^If not done, indicate that this assay was not performed.

The genetic background of animals can be described by species^18,19^, strain^9,20^, sub-strain^12^, breed/stock^21^ and breeding history (to trace contamination as an impact of the breeding scheme and genetic drift)^22^. Standards for their descriptions, such as the use of species-appropriate nomenclature conventions, are already defined. These are major intrinsic factors that have biological impact, and that must be fully documented to strengthen research reproducibility, complementing the ARRIVE guidelines. Table 1 summarizes the minimal information needed to correctly identify species and lineages. Criteria 1–5 report on information that is part of animal records in the laboratory. Criterion 6 reports the documentation of genetic assays that have recently become more accessible, both practically and financially, for many animal species. This last item validates the information provided in items 1–5. Examples of documentation are shown in Supplementary Table 1.

The genetic alterations carried by laboratory animals can affect phenotypes and require documentation. However, they are rarely fully documented, for both technical and historical reasons. Naturally occurring alleles or mutations obtained by chemical mutagenesis (e.g., ENU) require significant work to be defined^23–26^. Alleles obtained by gene targeting in embryonic stem (ES) cells are typically described by a schematic, with the full sequence of the targeting event rarely provided (with the particular exception of alleles that are produced by some high-throughput programs). Genome editing requires careful validation (including by sequencing) of the resulting allele (for example^27^), but documentation of the sequence of the entire region of interest is typically not provided. Furthermore, a consensus on the criteria for molecular validation of genetically engineered animals is yet to be defined. This is of particular importance because methods for both generation and validation of mutations are evolving rapidly, and new methods to validate larger regions of interest and identify both discrete and structural variations in genomes are emerging (for example, refs. ^28–30^). Of as much importance as the genetic background, the documentation of genetic alteration(s) and their validation represent essential areas for improvement in research reproducibility. The second part of Table 1 presents a set of criteria that should be used to describe genetic alterations in laboratory animals, which includes information about experimental design and confirmatory validation data. Examples of documentation for a genetically altered line and a checklist to support reviewers in assessing the documentation of genetics are shown in Supplementary Tables 1 and 2, respectively.

## Fundamentals of genetic validation for animal models

Validation of genetics refers to the verification of the overall genome for all animal models and, in the case of genetically altered animals, to the characterization of a specific region of interest. In some instances, the initial step will be to ascertain the most accurate taxonomy of the model at hand. In other contexts, the aim will be to check the pedigree of the stock. Specifically, inbred and outbred models present different challenges, as inbreeding aims to maintain genetic stability, whereas outbreeding aims to maintain genetic diversity^31^. Variations can be discrete alterations or structural changes that are likely to accumulate over time, and which should be identified and/or managed whenever possible.

### Genomic stability and quality

The genomes of animals change over their lifetime^32^ and with each generation^33,34^: both the accumulation of natural mutations (with fixation of variations resulting in genetic drift) and the modification of allelic diversity as a result of crossing patterns (inbreeding for outbred lines or contamination of lines by other backgrounds) could affect the genome composition of a laboratory animal^31^. For example, MMRRC UNC has performed a preliminary analysis of 230 lines, and they have estimated that approximately 40% of these lines do not match their name. The most common discrepancies are lack of congenicity, inbreeding, or the presence of additional genetic backgrounds (information from F.P.M.V., MMRRC). In addition, contaminating transgenes or unexpected altered alleles are also observed but at a low frequency. Good documentation and breeding strategies play an essential role in managing the quality of the genetic background of laboratory animals^35,36^ (Table 1), but additional techniques for capturing genetic variation are becoming available and affordable for many animal models (Supplementary Table 3). In particular, the Mouse Universal Genotyping Array (MUGA) panels can be used to verify the presence of many commonly used constructs, as well as to corroborate the composition of the genetic background of mice^22^. Similar panels are available for other species^37,38^. Similarly, quantitative polymerase chain reaction (qPCR) or digital polymerase chain reaction (dPCR) assays can be designed to detect common constructs used in a field of research or in a particular species. With the development of new technologies to evaluate genetic quality, a full genome assessment (including an understanding of the frequency of both discrete and structural variations) will become more accessible. Different laboratory animal species and scientific questions call for a different depth of genetic validation. However, in all cases, the more complete the validation of the genetics of the animal models, the more reliable and reproducible the experiments will be. Advances in the understanding and documentation of genetic variation do not prevent the occurrence of genetic changes during animal breeding but they allow researchers to monitor such changes and to re-evaluate phenotypes with the knowledge of newly described causative or modifying variants^39^. However, although genetic control is complementary to good practices in animal colony management, it has limited power when downstream crosses are made without rigor. Finally, many other factors affect the reproducibility of a given experiment, and a number of these are already covered in the ARRIVE guidelines^6^.

For a small number of laboratory animal species, advanced frameworks designed to manage their genetics already exist, mainly as a result of the length of time and the context in which they have been studied. These frameworks include knowledge of the species’ sequences and pedigrees, and support structures dedicated to maintaining genetic integrity. In those cases, reporting, traceability, and control of the genetic background of animals are even more essential. Where possible, crossing with wild-type reference animals is good practice to reduce de novo variations and construct contaminations within the genome. For example, current practice in mice is to backcross for two or three generations, depending on the level of inbreeding (https://www.jax.org/news-and-insights/jax-blog/2018/April/how-to-refresh-your-mutant-or-transgenic-mouse-strains^40^). Likewise, to avoid inbreeding depression in zebrafish stocks, each new generation should be produced by an outcross, and crosses between siblings should be performed only when absolutely necessary^41–43^. Although practices may vary across research communities and as fields evolve, the traceability of breeding patterns remains important in all circumstances.

Furthermore, all genome engineering techniques have the potential to introduce additional genetic changes while modifying the region of interest: both chromosome number and structure vary in embryonic stem (ES) cells when cultured^44^; both gene targeting and genome editing have the potential to insert additional copies of the donor template away from the target^45^; random insertion of DNA (transgenics) can introduce structural variation at the site of insertion^46^; and nucleases used in genome engineering activities are not entirely specific and can cause off-target variation^47^, though this is rare when specific design practices are used and must be evaluated in the context of known variation in the animals being studied^48^. Techniques have also been developed to identify these unwanted events^49,50^. These are discussed in the context of genome engineering validation in the next section.

### Assessing the genetic alterations

Genetic engineering was once restricted to a limited number of laboratory animal species^33^, but as a result of advances in genome-editing techniques, there is now almost no limit to the species that can be genetically engineered. Standards for validation are evolving in parallel with the technology.

Different modes of alteration have differing potential for unwanted outcomes. For example, whereas random insertions are the aim of additive transgenesis, other engineering technologies aim to target a specific locus. Therefore, no universal recommendation can be made for the validation of a genetic modification. When a specific locus is targeted, validation should aim to characterize the genetic change at the region of interest. In all cases, genetic changes resulting from the engineering method should be assessed throughout the genome. As for maintaining genomic stability and quality, multiple crosses to the reference genetic background will mitigate the potential genome-wide impact of genetic engineering and should be reported (Table 1). For targeted events, both the sequence of the region of interest and the local structural integrity should be examined, the latter for exclusion of deletion, duplication, and inversion events. Supplementary Table 4 lists the various molecular assays that can be employed to interrogate these two aspects. Ideally, genetic quality would be regularly assayed, but importing or onboarding animals is the most important point at which to check the quality of newly acquired or generated models. A combination of methods that elucidate both the sequence and structure of the target locus and the genetic makeup of samples (Mendelian, mosaic, or chimeric animals) is required. The methods used will depend on a number of factors, such as the laboratory setup, whether a donor sequence was used in the mutagenesis process and the length of the modified segment. For example, point mutations are easily characterized using Sanger or next-generation short-read (NGS) sequencing, whereas verification of very large knock-ins is likely to require a long-read-based sequencing approach. The functional characterization of the products of mutated genes is also an important aspect of research quality but is outside the scope of these recommendations.

### Further genome validation following genome engineering

All mutagenesis techniques have the potential to generate unpredictable and/or additional changes in the genome, outside of any region of interest, and these can be transmitted through generations. These may be discrete mutations^51^, additions, insertions^52^, or structural rearrangements (including chromothripsis^53,54^), in addition to naturally occurring genetic variation, as discussed above. It is essential to be aware of the occurrence of these nonconforming events to ensure the correct interpretation of results and research quality. A number of technologies and simple assays can be employed to screen animals for the presence of off-target events (including random integrations, which can be detected by dPCR), and these are summarized in Supplementary Table 5. However, some molecular changes have no recognizable pattern, meaning that no specific genotyping assay can be designed, or may affect difficult-to-characterize features, such as large segments or repeated sequences. Changes of this type will, therefore, require more sophisticated investigations, such as next-generation sequencing (Supplementary Table 5). No single technology yet allows for the unbiased and complete acquisition of the sequence of a whole genome^47,55^.

## The role of supporting frameworks for reporting animal genetics

There are numerous supporting frameworks for standardization initiatives that facilitate the knowledge and management of the genetic quality of laboratory animals. These include nomenclature guidelines, as well as repositories of information (such as ontologies, research data, metadata, and annotations) and materials.

Advanced systems of nomenclature are continuously being developed to describe animals and genes in a standardized fashion. Taxonomy resources include the National Center for Biotechnology Information taxonomy database^56^. In addition, the Vertebrate Gene Nomenclature Committee assigns standardized names to genes in vertebrate species that currently lack a nomenclature committee, ensuring that genes are named in line with their human orthologs^57,58^. These resources are essential to develop a common and unambiguous vocabulary with which to name genetic models and characteristics. They support the continuous refinement of nomenclature systems in sync with the evolution of animal models and molecular tools so that nomenclature remains compatible with state-of-the-art research.

The use of most laboratory models is supported by dedicated databases that aggregate genetic and phenotypic information and that link to other resources, such as sequencing databases, scientific publications, and animal model repositories (see examples in Supplementary Table 6). Researchers have a role to play in registering new animal models to publicly accessible databases, thus helping to avoid the generation of lines that already exist. Commercial breeders also distribute information on the biology of the animals they produce. Additional information with a focus on animal welfare can be collected through the establishment of identity cards^59^.

The integrity of genetic model materials is preserved through repositories that archive and distribute animals, gametes, and embryos, as well as plasmids and ES cells. These support structures are federated in international networks that collaborate to ensure the availability of quality-controlled materials to the research community worldwide. The collections available in these repositories can be interrogated at their individual web portals or through web pages that allow querying of the entire repository network to locate and source animal models (https://www.alliancegenome.org/^4^). Together with academic and commercial research animal breeders, these repositories play a crucial role in ensuring the genetic quality and stability of laboratory animals and the reproducibility of research that employs animal models.

Acquiring knowledge of appropriate standards of documentation, with the ability to understand and employ these, is an integral part of scientific training. This includes a knowledge of genetics. Beyond formal education, many web resources and training opportunities are available (e.g., https://oacu.oir.nih.gov/training-resources; https://www.aalas.org/iacuc/iacuc_resources/training-programs; https://resources.jax.org/). In this respect, learned societies, breeders, and repositories of laboratory animals are important sources of information and educational material.

Finally, the FAIRsharing portal aims to aggregate the resources that support standardization in the life sciences (https://fairsharing.org^7^). Likewise, learned societies and dedicated consortia play an essential role in establishing these research-support frameworks and in facilitating the training of researchers to understand and manage the challenges of using laboratory animals for reproducible research.

## Perspectives

Recognizing concerns about reproducibility, the LAG-R guidelines aim to standardize the information about animal models in scientific reports. This is becoming increasingly important as the diversity of laboratory animals expands, along with new methods and designs for the generation of genetic alterations and for in-depth characterization of genomes. However, we must not ignore that there are barriers to overcome. In particular, it requires a consensus within the community, greater expertise in genetics, and additional editorial work on the part of authors, reviewers, and editors.

It still does not seem realistic, or even possible, to fully validate the entire genetic composition of every animal used in research. On the other hand, improved reporting of all available information regarding the genetic makeup of laboratory animal models and on which validations have been carried out will allow us to better reinforce and evaluate the reliability of animal experiments. More in-depth animal model validation is increasingly feasible but requires specific expertise and the availability of dedicated funding, two aspects that will require significant investment.

Going forward, it is for the community to improve laboratory animal genetic reporting and the LAG-R guidelines will help to facilitate this, but only with the commitment of scientists, funding bodies, journals, reviewers and editors.

### Supplementary information

Supplementary Information

## References

[1] Baker, M. 1,500 scientists lift the lid on reproducibility. *Nature* **533**, 452–454 (2016). 27225100 10.1038/533452a

[2] Lloyd, K., Franklin, C., Lutz, C. & Magnuson, T. Reproducibility: Use mouse biobanks or lose them. *Nature* **522**, 151–153 (2015). 26062496 10.1038/522151a PMC4636083

[3] Dessimoz, C., Škunca N. *The Gene Ontology Handbook* Vol. 1446 (Springer, New York, NY, 2017).

[4] Alliance of Genome Resources Consortium et al. Harmonizing model organism data in the Alliance of Genome Resources. *Genetics* **220**, iyac022 (2022). 35380658 10.1093/genetics/iyac022 PMC8982023

[5] Smith, A. J., Clutton, R. E., Lilley, E., Hansen, K. E. A. & Brattelid, T. PREPARE: guidelines for planning animal research and testing. *Lab. Anim.* **52**, 135–141 (2018). 28771074 10.1177/0023677217724823 PMC5862319

[6] Percie du Sert, N. et al. The ARRIVE guidelines 2.0: Updated guidelines for reporting animal research. *PLoS Biol.* **18**, e3000410 (2020). 32663219 10.1371/journal.pbio.3000410 PMC7360023

[7] the FAIRsharing Community et al. FAIRsharing as a community approach to standards, repositories and policies. *Nat. Biotechnol.* **37**, 358–367 (2019). 30940948 10.1038/s41587-019-0080-8 PMC6785156

[8] Percie du Sert, N. et al. Reporting animal research: explanation and elaboration for the ARRIVE guidelines 2.0. *PLoS Biol.* **18**, e3000411 (2020). 32663221 10.1371/journal.pbio.3000411 PMC7360025

[9] Sittig, L. J. et al. Genetic background limits generalizability of genotype–phenotype relationships. *Neuron* **91**, 1253–1259 (2016). 27618673 10.1016/j.neuron.2016.08.013 PMC5033712

[10] Doetschman, T. Influence of genetic background on genetically engineered mouse phenotypes. In *Gene Knockout Protocols* Vol. 530 (eds. Wurst, W. & Kühn, R.) 423–433 (Humana Press, Totowa, NJ, 2009). 10.1007/978-1-59745-471-1_23 PMC2805848 19266333

[11] Strobel, M. C., Reinholdt, L. G., Malcolm, R. D. & Pritchett-Corning, K. Genetic monitoring of laboratory mice and rats. In *Laboratory Animal Medicine*. (eds Fox, J. G. et al.) 1403–1416 (Elsevier, 2015).

[12] Simon, M. M. et al. A comparative phenotypic and genomic analysis of C57BL/6J and C57BL/6N mouse strains. *Genome Biol.* **14**, R82 (2013). 23902802 10.1186/gb-2013-14-7-r82 PMC4053787

[13] Voelkl, B. et al. Reproducibility of animal research in light of biological variation. *Nat. Rev. Neurosci.* **21**, 384–393 (2020). 32488205 10.1038/s41583-020-0313-3

[14] Zeldovich, L. Genetic drift: the ghost in the genome. *Lab Anim.* **46**, 255–257 (2017). 10.1038/laban.1275 28530691

[15] Freedman, L. P., Cockburn, I. M. & Simcoe, T. S. The economics of reproducibility in preclinical research. *PLoS Biol.* **13**, e1002165 (2015). 26057340 10.1371/journal.pbio.1002165 PMC4461318

[16] Jacquot, S., Chartoire, N., Piguet, F., Hérault, Y. & Pavlovic, G. Optimizing PCR for mouse genotyping: recommendations for reliable, rapid, cost effective, robust and adaptable to high‐throughput genotyping protocol for any type of mutation. *Curr. Protoc. Mouse Biol.* **9**, e65 1–28 (2019). 10.1002/cpmo.65 31756054

[17] Russell, W. M. S. & Burch, R. L. *The Principles of Humane Experimental Technique*. (Methuen, 1959).

[18] Engle, S. HPRT-APRT-deficient mice are not a model for Lesch–Nyhan syndrome. *Hum. Mol. Genet.* **5**, 1607–1610 (1996). 8894695 10.1093/hmg/5.10.1607

[19] Meek, S. et al. Reduced levels of dopamine and altered metabolism in brains of HPRT knock-out rats: a new rodent model of Lesch–Nyhan Disease. *Sci. Rep.* **6**, 25592 (2016). 27185277 10.1038/srep25592 PMC4869022

[20] Bilovocky, N. A., Romito-DiGiacomo, R. R., Murcia, C. L., Maricich, S. M. & Herrup, K. Factors in the genetic background suppress the *Engrailed-1* cerebellar phenotype. *J. Neurosci.* **23**, 5105–5112 (2003). 12832534 10.1523/JNEUROSCI.23-12-05105.2003 PMC6741147

[21] Axelsson, E. et al. The genetic consequences of dog breed formation—accumulation of deleterious genetic variation and fixation of mutations associated with myxomatous mitral valve disease in cavalier King Charles spaniels. *PLoS Genet.* **17**, e1009726 (2021). 34473707 10.1371/journal.pgen.1009726 PMC8412370

[22] Sigmon, J. S. et al. Content and performance of the MiniMUGA genotyping array: a new tool to improve rigor and reproducibility in mouse research. *Genetics* **216**, 905–930 (2020). 33067325 10.1534/genetics.120.303596 PMC7768238

[23] Barbaric, I. et al. An ENU-induced mutation in the Ankrd11 gene results in an osteopenia-like phenotype in the mouse mutant Yoda. *Physiol. Genom.* **32**, 311–321 (2008). 10.1152/physiolgenomics.00116.2007 17986521

[24] De Angelis, M. H. et al. Genome-wide, large-scale production of mutant mice by ENU mutagenesis. *Nat. Genet.* **25**, 444–447 (2000). 10932192 10.1038/78146

[25] Andersson, L. Molecular consequences of animal breeding. *Curr. Opin. Genet. Dev.* **23**, 295–301 (2013). 23601626 10.1016/j.gde.2013.02.014

[26] Ciepłoch, A., Rutkowska, K., Oprządek, J. & Poławska, E. Genetic disorders in beef cattle: a review. *Genes Genom.* **39**, 461–471 (2017). 10.1007/s13258-017-0525-8 PMC5387086 28458779

[27] Bunton-Stasyshyn, R. K., Codner, G. F. & Teboul, L. Screening and validation of genome-edited animals. *Lab Anim.* **56**, 69–82 (2022). 34192966 10.1177/00236772211016922 PMC9008557

[28] Marx, V. Method of the year: long-read sequencing. *Nat. Methods* **20**, 6–11 (2023). 36635542 10.1038/s41592-022-01730-w

[29] De Coster, W. & Van Broeckhoven, C. Newest methods for detecting structural variations. *Trends Biotechnol.* **37**, 973–982 (2019). 30902345 10.1016/j.tibtech.2019.02.003

[30] Chan, S. et al. Structural variation detection and analysis using bionano optical mapping. In *Copy Number Variants* Vol. 1833 (ed. Bickhart, D. M.) 193–203 (Springer, New York, 2018). 10.1007/978-1-4939-8666-8_16 30039375

[31] Benavides, F. et al. Genetic quality assurance and genetic monitoring of laboratory mice and rats: FELASA Working Group Report. *Lab. Anim.* **54**, 135–148 (2020). 31431136 10.1177/0023677219867719 PMC7160752

[32] Cagan, A. et al. Somatic mutation rates scale with lifespan across mammals. *Nature* **604**, 517–524 (2022). 35418684 10.1038/s41586-022-04618-z PMC9021023

[33] Milholland, B. et al. Differences between germline and somatic mutation rates in humans and mice. *Nat. Commun.* **8**, 15183 (2017). 28485371 10.1038/ncomms15183 PMC5436103

[34] Lynch, M. Evolution of the mutation rate. *Trends Genet.* **26**, 345–352 (2010). 20594608 10.1016/j.tig.2010.05.003 PMC2910838

[35] Fox, J.G. et al. *The Mouse in Biomedical Research* (Elsevier, Amsterdam; Boston, 2007).

[36] Rogers, J. Genomic resources for rhesus macaques (*Macaca mulatta*). *Mamm. Genome* **33**, 91–99 (2022). 34999909 10.1007/s00335-021-09922-z PMC8742695

[37] Vaysse, A. et al. Identification of genomic regions associated with phenotypic variation between dog breeds using selection mapping. *PLoS Genet.* **7**, e1002316 (2011). 22022279 10.1371/journal.pgen.1002316 PMC3192833

[38] Matsuda, K. PCR-based detection methods for single-nucleotide polymorphism or mutation. In *Advances in Clinical Chemistry* Vol. 80 45–72 (Elsevier, 2017). 10.1016/bs.acc.2016.11.002 28431642

[39] Rawle, D. J. et al. Widespread discrepancy in Nnt genotypes and genetic backgrounds complicates granzyme A and other knockout mouse studies. *eLife* **11**, e70207 (2022). 35119362 10.7554/eLife.70207 PMC8816380

[40] Kelmenson, P. *How to Refresh Your Mutant or Transgenic Mouse Strains* https://www.jax.org/news-and-insights/jax-blog/2018/april/how-to-refresh-your-mutant-or-transgenic-mouse-strains (2018).

[41] Trevarrow, B. & Robison, B. Genetic backgrounds, standard lines, and husbandry of zebrafish. *Methods Cell Biol.* **77**, 599–616 (2004). 15602934 10.1016/S0091-679X(04)77032-6

[42] Varga, Z. M. Aquaculture, husbandry, and shipping at the Zebrafish International Resource Center. *Methods Cell Biol.* **135**, 509–534 (2016). 27443942 10.1016/bs.mcb.2016.01.007

[43] Martins, S. et al. Toward an integrated zebrafish health management program supporting cancer and neuroscience research. *Zebrafish* **13**, S47–S55 (2016). 26959533 10.1089/zeb.2015.1198

[44] Liang, Q., Conte, N., Skarnes, W. C. & Bradley, A. Extensive genomic copy number variation in embryonic stem cells. *Proc. Natl Acad. Sci. USA* **105**, 17453–17456 (2008). 18988746 10.1073/pnas.0805638105 PMC2582305

[45] Lintott, L. G. & Nutter, L. M. J. Genetic and Molecular Quality Control of Genetically Engineered Mice. In *Transgenesis* Vol. 2631 (ed. Saunders, T. L.) 53–101 (Springer US, New York, NY, 2023). 10.1007/978-1-0716-2990-1_3 36995664

[46] Goodwin, L. O. et al. Large-scale discovery of mouse transgenic integration sites reveals frequent structural variation and insertional mutagenesis. *Genome Res.* **29**, 494–505 (2019). 30659012 10.1101/gr.233866.117 PMC6396414

[47] Burgio, G. & Teboul, L. Anticipating and Identifying Collateral Damage in Genome Editing. *Trends Genet.* **36**, 905–914 (2020). 33039248 10.1016/j.tig.2020.09.011 PMC7658041

[48] Peterson, K. A. et al. Whole genome analysis for 163 gRNAs in Cas9-edited mice reveals minimal off-target activity. *Commun. Biol.* **6**, 626 (2023). 37301944 10.1038/s42003-023-04974-0 PMC10257658

[49] Anderson, K. R. et al. CRISPR off-target analysis in genetically engineered rats and mice. *Nat. Methods* **15**, 512–514 (2018). 29786090 10.1038/s41592-018-0011-5 PMC6558654

[50] Manghwar, H. et al. CRISPR/Cas systems in genome editing: methodologies and tools for sgRNA design, off‐target evaluation, and strategies to mitigate off‐target effects. *Adv. Sci.* **7**, 1902312 (2020). 10.1002/advs.201902312 PMC7080517 32195078

[51] CRISPR off-targets: a reassessment. *Nat Methods* **15**, 229–230 (2018).

[52] Norris, A. L. et al. Template plasmid integration in germline genome-edited cattle. *Nat. Biotechnol.* **38**, 163–164 (2020). 32034391 10.1038/s41587-019-0394-6

[53] Leibowitz, M. L. et al. Chromothripsis as an on-target consequence of CRISPR–Cas9 genome editing. *Nat. Genet.* **53**, 895–905 (2021). 33846636 10.1038/s41588-021-00838-7 PMC8192433

[54] Bertelsen, B. et al. A germline chromothripsis event stably segregating in 11 individuals through three generations. *Genet. Med.* **18**, 494–500 (2016). 26312826 10.1038/gim.2015.112

[55] Nurk, S. et al. The complete sequence of a human genome. *Science* **376**, 44–53 (2022). 35357919 10.1126/science.abj6987 PMC9186530

[56] Schoch, C. L. et al. NCBI Taxonomy: a comprehensive update on curation, resources and tools. *Database* **2020**, baaa062 (2020). 32761142 10.1093/database/baaa062 PMC7408187

[57] Tweedie, S. et al. Genenames.org: the HGNC and VGNC resources in 2021. *Nucleic Acids Res.* **49**, D939–D946 (2021). 33152070 10.1093/nar/gkaa980 PMC7779007

[58] McCarthy, F. M. et al. The case for standardizing gene nomenclature in vertebrates. *Nature* **614**, E31–E32 (2023). 36792746 10.1038/s41586-022-05633-w PMC9931569

[59] Wells, D. J. et al. Assessing the welfare of genetically altered mice. *Lab Anim.* **40**, 111–114 (2006). 16600070 10.1258/002367706776318971

[60] Lalonde, R., Filali, M. & Strazielle, C. SHIRPA as a neurological screening battery in mice. *Curr. Protoc.* **1**, e135 1–30 (2021). 10.1002/cpz1.135 34000103

[61] Patange, S. & Maragh, S. Fire burn and cauldron bubble: what is in your genome editing brew? *Biochemistry*10.1021/acs.biochem.2c00431 (2022). 10.1021/acs.biochem.2c00431 PMC10734218 36306429

