# Supplementary material for: Improving laboratory animal genetic reporting: LAG-R guidelines
Source: Nat Commun. 2024 Jul 2;15:5574. doi: 10.1038/s41467-024-49439-y (PMC11220107; doi:10.1038/s41467-024-49439-y)
Supplement: Supplementary file 1 — Supplementary Information [file 41467_2024_49439_MOESM1_ESM.pdf]

Supplementary Information - Improving laboratory animal genetic reporting: LAG-R guidelines

Supplementary Tables

Supplementary Table 1: Examples of the use of LAG-R recommendations for a rodent model and a zebrafish model

Two examples are provided. The first one, in mouse, is based on Mianne *et al* [1]. The second one, in zebrafish, is based on Hayot *et al* [2].

**CRITERIA TO REPORT ON FOR ALL RESEARCH ANIMALS**[1]			**EXAMPLE IN MOUSE**	**EXAMPLE IN ZEBRAFISH**
Genetic background description including Strain/breed/stock type	1	**Official name of species, strain, and sub-strain, as applicable, of the animal. Alternatively, for farm animals, indicate breed https://www.ncbi.nlm.nih.gov/Taxonomy/Browser/wwwtax.cgi.** **Describe type of breeding for strain/stock/breed using species standard wording.** **For example, in rodent: outbred, inbred, hybrid, congenic**[2]**, documented mixed or non-documented genetic background. For example, in zebrafish: specify which wildtype background (AB, TU, TL); if mixed background, provide a clear explanation of the breeding scheme and estimation of percentage of each background.**	*Mus musculus,* C57BL/6NTac substrain Mutagenized embryos are described in Materials and Methods. Stock used for germ line transmission are described in Results and Discussion.	*Danio rerio*, AB strain Maintenance of stock, fish stock density, water, temperature, dark-light cycle, water chemistry and fish feed and feeding frequency are described in Materials and Methods. Mutant zebrafish line and origin of wildtype line utilized to maintain genetic quality of the colony are described in Materials and Methods.
Breeding scheme and stability program (only relevant for rodents)[3]	2	**Specify breeding schemes used to maintain stock and generate experimental animals. Include the genotype of the parents when possible. This is particularly important to trace the origin of sex chromosomes in congenic strains**[2]**.** *Specify breeding strategy to maintain genetic quality of the colony; indicate known family tree.*	Heterozygous *Cdh23*^{em1H} mice were backcrossed onto C57BL/6NTac.	Heterozygous mutant zebrafish were backcrossed onto AB strain obtained from the European Zebrafish Resource Center (EZRC, stock number #24433).
Source of animals	3	**Name origin of strain(s). Name supplier or repository or other origin of animals used in the experiment.**	The Mary Lyon Centre at MRC Harwell	EZRC
International nomenclature	4	**Name strain according to internationally agreed standard when available.**[3] *Use research resource identifier (RRID) when applicable.*	C57BL/6NTac-*Cdh23*^{em1H}/*H*	*chd8*^{sa19827} (TL)
Strain or stock identifier	5	*Show unique identifier of strain or stock used by the supplier or the repository.*	EM:11508	EZRC#24433

Genetic background validation	6	*Indicate if, when (at what breeding generation) and how the genetic background was verified (i.e., sequencing, SNP (single-nucleotide polymorphism) panel, STR panel, genetic testing chip panel).*	F1 animals were verified using the SNP panel described in Supplementary Table 7 (in this document, not Supplemental from initial paper)	N/A

ADDITIONAL CRITERIA TO REPORT ON FOR ANIMALS WITH GENETIC ALTERATION[1]			EXAMPLE IN MOUSE	EXAMPLE IN ZEBRAFISH
Name of mutant allele	7	**Detail the shorthand used in article, and official nomenclature.[4]** *Use unique identifier (e.g. MGI ID) when applicable.*	Shorthand in the publication is *Cdh23*$^{753A>G}$; MGI: 5661070; official nomenclature is *Cdh23*$^{ahl+em1H}$	Shorthand in the publication is *chd8*^{C667T}; EZRC: #24433; ID: ZDB-ALT-160601-32; official nomenclature is *chd8*^{sa19827}
Allele type	8	**Specify the method of model creation: naturally occurring allele/gene targeting/genome editing/additive transgenesis/chemical or physical mutagenesis/viral insertion/site-specific recombination/transposition.**	Genome editing in one cell stage embryo. Materials & Methods describes in detail how the genome editing experiments were performed.	Adult males treated with ENU
Intended and observed consequence of mutagenesis	9	*Detail whether allele is a frameshift, deletion, coding or non-coding variant, overexpression, conditional allele, humanization, reporter, structural variation. Detail new gene product if known.*	This mutation reinforces the splice donor sequence mutated in C57BL/6N mice.	The mutation leads to a premature stop codon.
Model summary description	10	**Provide a short summary of genetic modification and background used for establishing genetic alteration.**	A short summary as provided by MGI.<br>The CRISPR/Cas9 system was used to correct the Cdh23^{ahl} allele (c.753A>G). This engineered point mutation mimics the C variant of SNP rs257098870 (G on negative gene strand) that is found in the 129S1/SvImJ, C3H/HeSnJ, I/LnJ, YBR/Ei, and MRL/MpJ strains. It restores the G-GT splice donor site that has changed to A-GT in C57BL/6 (and most other strains), where it causes skipping of in-frame exon 7. (*J:90559*, *J:236297*).<br>Mutation was introduced in C57BL/6NTac mice.	Adult males from TL background were treated with ENU in the Stemple Lab. The mutant line carries the point mutation c.C667T (p.Glu223*) at position: Chr 2: 38115067 (GRCz11). The mutation leads to a premature stop. Information on the mutant line can be found on ZFIN at http://zfin.org/ZDB-ALT-160601-32#summary.
DNA sequence	11	**Provide access to the sequence of the genetic modification: targeting vector, donor template or vector for transgenesis. If employed in the mutagenesis process, provide the sequence of donor (e.g., targeting vector, oligodeoxynucleotide, transgene or template sequence used for mutagenesis; DNA or prime editing guide).[5]** *Annotate genomic sequences with corresponding genome assembly version and coordinates. Use universal format; i.e., .fasta or .gb. Annotate features.*	The sequence of the genetic modification corresponds to the sequence of the donor template. Sequence of one representative F1 animal is presented in Figure 2.<br>Supplementary Table S1 contains the sequences of the donor.	Position of the mutation and annotation can be found on ZFIN at http://zfin.org/ZDB-ALT-160601-32#summary.
Allele schematic	12	*Consider presenting a map of the genetic modification.*	Described in Figure 1.	Available on ZFIN. http://zfin.org/ZDB-ALT-160601-32#summary.
Material availability/source of materials	13	**Describe how to access available materials (plasmids, mutant cells, animals and/or germplasm).** *RRID and/or repository identifier.*	Animals and/or germplasm is accessible through the Infrafrontier repository; repository identifier: EM:11508. Plasmids are available through contacting MRC Harwell.	The mutant line is available at EZRC (#24433) and ZIRC (Catalog ID. ZL12033.03)
Obvious phenotype and welfare concern	14	**Specify salient phenotypes, such as issues with viability and/or fertility, or immunodeficiency. Describe the severity**	Age-related hearing loss is corrected by the mutations. No welfare issues are associated with this mutation.	All fish lines reproduce normally, andchd8 homozygote mutants were recovered in the expected Mendelian

		**of the associated phenotype. If necessary, include any requirement to mitigate welfare concerns. Include publication, archive or database reference if available.** *For mice, consider SHIRPA (SmithKline Beecham/Harwell/Imperial College/Royal London Hospital/phenotype assessment) description (supp ref 3).*		ratio. A skewed sex ratio was observed with almost only males.
Initial reference	15	**Detail whether report is the initial description of mutant and/or mention initial publication of materials.**	This paper is the initial reference. PMID: 26876963	This paper is the initial reference. PMID: 36375841
Genotyping assay	16	**Describe assay and sequence of primers used for genotyping of established colony.**	The animals from the established colony are genotyped using an allelic discrimination assay run according to Kamau et al., 2012) with the following primers:<br>Cdh23 F Primer: GACATGGATCCTATCTTCATCAACC<br>Cdh23 R Primer: CCAGCACGGGCTAGAGAAC<br>Wild-type Allele probe (FAM-Labelled): CACTCTCCTCC**A**GTGAG<br>Mutant Allele probe (TET-Labelled): CTCCTCC**G**GTGAGC	The animals were genotyped as described in Materials and Methods and by utilizing the following primers:<br>Chd8 F primer GTCAGACTCAAGTGCTGCAG<br>Chd8 R primer GACACTTTGGTCGGAT<br>The PCR product was digested by the *RsaI* enzyme, a restriction enzyme whose restriction site is disrupted by the sa19827 mutation.
Enzyme and other reagents used for genome engineering	17	**Describe enzymes (nuclease, recombinase) if used to generate mutation including number and sequence of guide(s) for ribonucleoproteins if relevant. Detail reagents.[6]**	Materials & Methods describes the genome editing reagents used to generate the model including the origin of Cas9 sequence used.<br>Supplementary Table S1 contains the sequences of the guides.	*N/A*
Validation of allele sequence	18	*If done, describe how the region of interest was validated.[7]*	See Figure 2 and Results.	*N/A*
Validation of allele structure	19	*If done, describe the precise method used for validation of chromosomal or allele structure, and the outcome.[7]*	This experiment was not performed. The depth of whole genome sequencing performed did not allow estimation of the structural variation.	*N/A*
Validation to exclude additional integration of mobilized sequence	20	*If done, describe the method and outcome of analyzing the material for additional integrations of donor templates[5] or reintegration of deleted segments.[7]*	Copy counting of ssODN is described in Materials & Methods. Whole genome sequencing is described in the Results section.	*N/A*
Evaluation of potential off-target activity	21	*Genome editing off-target is defined as a genomic position and/or nucleic acid sequence distinct from the target. If done, describe the method, selection criteria and outcome of off-target analysis.*	Off-target analysis is described in the Results and Supplemental sections.	*N/A*

Note 1. Essential criteria are indicated in bold; recommended criteria are indicated in italic. The information itself, or a reference to a source, should be detailed.

Note 2. Congenic strains are examples of the importance of correct breeding scheme, as their genetic composition varies according to the parental origin of the sex chromosomes and the mitochondrial genome.

In addition, the identity of the region around a transmitted allele remains that of the original strain.

Note 3. Definitions and guidelines for nomenclature of mouse and rat strains are described at https://www.informatics.jax.org/mgihome/nomen/strains.shtml

Note 4. Guidelines for Nomenclature of Genes, Genetic Markers, Alleles, and Mutations in Mouse and Rat are described at https://www.informatics.jax.org/mgihome/nomen/gene.shtml.

Note 5. Note that donor sequence can differ from mutagenesis outcome.

Note 6. Some recommendations for genome-editing formulations are reported in supp ref 4.

Note 7. If not done, indicate that this assay was not performed.

Supplementary Table 2: LAG-R checklist for reviewers

**CRITERIA TO REPORT ON FOR ALL RESEARCH ANIMALS**[1]			**checkbox**
Genetic background description including Strain/breed/stock type	1	**Official name of species, strain, and sub-strain, as applicable, of the animal. Alternatively, for farm animals, indicate breed https://www.ncbi.nlm.nih.gov/Taxonomy/Browser/wwwtax.cgi. Describe type of breeding for strain/stock/breed using species standard wording. For example, in rodent: outbred, inbred, hybrid, congenic[2], documented mixed or non-documented genetic background. For example, in zebrafish: specify which wildtype background (AB, TU, TL); if mixed background, provide a clear explanation of the breeding scheme and estimation of percentage of each background.**	☐
Breeding scheme and stability program (only relevant for rodents)[3]	2	**Specify breeding schemes used to maintain stock and generate experimental animals. Include the genotype of the parents when possible. This is particularly important to trace the origin of sex chromosomes in congenic strains[2].** *Specify breeding strategy to maintain genetic quality of the colony; indicate known family tree.*	☐
Source of animals	3	**Name origin of strain(s). Name supplier or repository or other origin of animals used in the experiment.**	☐
International nomenclature	4	**Name strain according to internationally agreed standard when available.[3]** *Use research resource identifier (RRID) when applicable.*	☐
Strain or stock identifier	5	*Show unique identifier of strain or stock used by the supplier or the repository.*	☐
Genetic background validation	6	*Indicate if, when (at what breeding generation) and how the genetic background was verified (i.e., sequencing, SNP (single-nucleotide polymorphism) panel, STR panel, genetic testing chip panel).*	☐
**ADDITIONAL CRITERIA TO REPORT ON FOR ANIMALS WITH GENETIC ALTERATION**[1]			
Name of mutant allele	7	**Detail the shorthand used in article, and official nomenclature.[4]** *Use unique identifier (e.g. MGI ID) when applicable.*	☐
Allele type	8	**Specify the method of model creation: naturally occurring allele/gene targeting/genome editing/additive transgenesis/chemical or physical mutagenesis/viral insertion/site-specific recombination/transposition.**	☐
Intended and observed consequence of mutagenesis	9	*Detail whether allele is a frameshift, deletion, coding or non-coding variant, overexpression, conditional allele, humanization, reporter, structural variation. Detail new gene product if known.*	☐
Model summary description	10	**Provide a short summary of genetic modification and background used for establishing genetic alteration.**	☐
DNA sequence	11	**Provide access to the sequence of the genetic modification: targeting vector, donor template or vector for transgenesis. If employed in the mutagenesis process, provide the sequence of donor (e.g., targeting vector, oligodeoxynucleotide, transgene or template sequence used for mutagenesis; DNA or prime editing guide).[5]** *Annotate genomic sequences with corresponding genome assembly version and coordinates. Use universal format; i.e., .fasta or .gb. Annotate features.*	☐
Allele schematic	12	*Consider presenting a map of the genetic modification.*	☐
Material availability/source of materials	13	**Describe how to access available materials (plasmids, mutant cells, animals and/or germplasm).** *RRID and/or repository identifier.*	☐
Obvious phenotype and welfare concern	14	**Specify salient phenotypes, such as issues with viability and/or fertility, or immunodeficiency. Describe the severity of the associated phenotype. If necessary, include any requirement to mitigate welfare concerns. Include publication, archive or database reference if available.** *For mice, consider SHIRPA (SmithKline Beecham/Harwell/Imperial College/Royal London Hospital/phenotype assessment) description (supp ref 3).*	☐
Initial reference	15	**Detail whether report is the initial description of mutant and/or mention initial publication of materials.**	☐

Genotyping assay	16	**Describe assay and sequence of primers used for genotyping of established colony.**	☐
Enzyme and other reagents used for genome engineering	17	**Describe enzymes (nuclease, recombinase) if used to generate mutation including number and sequence of guide(s) for ribonucleoproteins if relevant. Detail reagents.[6]**	☐
Validation of allele sequence	18	*If done, describe how the region of interest was validated.[7]*	☐
Validation of allele structure	19	*If done, describe the precise method used for validation of chromosomal or allele structure, and the outcome.[7]*	☐
Validation to exclude additional integration of mobilized sequence	20	*If done, describe the method and outcome of analyzing the material for additional integrations of donor templates[4] or reintegration of deleted segments.[7]*	☐
Evaluation of potential off-target activity	21	*Genome editing off-target is defined as a genomic position and/or nucleic acid sequence distinct from the target. If done, describe the method, selection criteria and outcome of off-target analysis.*	☐

Note 1. Essential criteria are indicated in bold; recommended criteria are indicated in italic. The information itself, or a reference to a source, should be detailed.

Note 2. Congenic strains are examples of the importance of correct breeding scheme, as their genetic composition varies according to the parental origin of the sex chromosomes and the mitochondrial genome. In addition, the identity of the region around a transmitted allele remains that of the original strain.

Note 3. Definitions and guidelines for nomenclature of mouse and rat strains are described at https://www.informatics.jax.org/mgihome/nomen/strains.shtml

Note 4. Guidelines for Nomenclature of Genes, Genetic Markers, Alleles, and Mutations in Mouse and Rat are described at https://www.informatics.jax.org/mgihome/nomen/gene.shtml.

Note 5. Note that donor sequence can differ from mutagenesis outcome.

Note 6. Some recommendations for genome editing formulations are reported in supp ref 4.

Note 7. If not done, indicate that this assay was not performed.

Supplementary Table 3: Examples of techniques for the analysis of genetic background

**Techniques**	**Limitations**	**Reference**
SNP PCR/qPCR panel	Availability of a panel for the species/strain	5,6
Short tandem repeat (STR), simple sequence length polymorphism (SSLP), microsatellites	Availability of a panel for the species/strain	7–11
Chip panel		5,12
Overt phenotype		Obvious traits relevant to the species/strain/sub-strain/breed/stock (e.g. coat color) 13
Short and long read WGS (high depth)	May be complex in outbred context	14

Supplementary Table 4: Examples of methods for evaluation of targeted allele quality (sequence and structure)

**Methods for allele sequence**
PCR followed by Sanger sequencing
PCR; subcloning followed by sequencing
PCR followed by NGS[1]
PCR for long amplicons and targeted NGS sequencing
DNA enrichment (e.g., Samplix capture, Cas9 capture, adaptive sampling) with deep short- or long-read NGS sequencing[2]
**Methods for allele structure**
Southern blot
qPCR, dPCR
Microarray
Targeted NGS sequencing of long amplicons[1]
DNA enrichment (e.g., inverse PCR-based techniques such as Targeted Locus Amplification capture, Samplix, Cas9 capture, adaptive sampling) with long-read sequencing
FISH
Fiber-FISH

Note 1. Limited by PCR amplicon size.
Note 2. Depends on experimental design and read depth.

Supplementary Table 5: Examples of methods for evaluation of off-target genetic changes

**Additional insertion of donors**[1]	**Structural alterations**	**Discrete sequence alterations**
Southern blot	Southern blot	PCR & Sanger sequencing[2]
dPCR and qPCR	Optical genome mapping	High-throughput NGS analysis of PCR amplicons from algorithm-predicted off-target loci
Plasmid backbone PCR	G-banding	NGS WGS (high depth)
TLA and other inverse PCR based methods	FISH	
DNA capture (e.g. Samplix capture, nCATS) followed by deep short or long read NGS sequencing[3]	Fiber-FISH	
Chip panel	Short- and long-read WGS (high depth)	

Note 1. and other mobilized elements (e.g., deleted fragments)

Note 2. List of sites at higher risk for unintended edits can be bioinformatically estimated or experimentally estimated by a capture method (e.g., GUIDE-seq, CIRCLE-seq, LAM–HTGTS, UDiTaS™, Digenome-seq CHANGE-seq, DISCOVER-seq.)

Note 3. Depends on experimental design and read depth.

Supplementary Table 6: Examples of support for nomenclature relating to laboratory animals (Metazoa clade)

**Resource**	**Acronym**	**Species**	**Relevant website and resource**
International Commission on Zoological Nomenclature	ICZN	Animals	https://www.iczn.org/
Anolis Gene Nomenclature Committee	AGNC	*Anolis carolinensis*	[15]
National Association of Animal breeders	NAAB	*Bos taurus*	https://www.naab-css.org/uniform-breed-codes
Official Cattle Breeds and codes (British Cattle Movement Services)	BCMS	*Bos taurus*	https://www.gov.uk/guidance/official-cattle-breeds-and-codes
WormBase	N/A	*Caenorhabditis elegans*	https://wiki.wormbase.org/index.php/Nomenclature
Zebrafish Nomenclature Committee	ZNC	*Danio rerio*	https://zfin.org/zf_info/news/committees.html
FlyBase	N/A	*Drosophila melanogaster*	https://wiki.flybase.org/wiki/FlyBase:Nomenclature
Chicken Gene Nomenclature Committee	CGNC	*Gallus gallus domesticus*	[16]
International Committee on Standardized Genetic Nomenclature for Mice	ICSGNM	*Mus musculus*	https://www.informatics.jax.org/mgihome/nomen/
Rat Genome Nomenclature Committee	RGNC	*Rattus norvegicus*	https://rgd.mcw.edu/nomen/nomen.shtml
Xenopus Nomenclature Committee	XNC	*Xenopus sp.*	https://www.xenbase.org
Breeds of Livestock	N/A	N/A	https://breeds.okstate.edu/

Supplementary Table 7: Genetic background quality control for line C57BL/6NTac-Cdh23^{em1H}/H

Eight allelic discrimination assays that discriminate the C57BL/6NTac from the C57BL/6J backgrounds were run according to Kamau *et al* [17]. All probes contained a 3' BHQ-1 plus modification.

			**C57BL/6NTac**		**C57BL/6J**	
**Assay name**	**Forward sequence**	**Reverse primer sequence**	**Probe 1 5' modification**	**Probe 1 sequence**	**Probe 2 5' modification**	**Probe 2 sequence**
4_28.49	CTGAAGGTCTCCATTGCCACAT	GGCCATGCCAAACATTATCTGA	FAM	AGGGCTAGTGTGTCTTCTTG	TET	AGGGCTAGTGTGTTTTCTTG
6_67.21	ACTCTTAAAGCAGTCCTGTCACTT	GACAAAGAAAGACAGTAACACACAC	FAM	AAAGTGAGATGTCTATCAGGTA	TET	AAAGTGAGATGTCTTTCAGGT
10_57.79	TCATGTGCGCATTGTATGTGTG	GGTAGGTTGAGCGCCTATCTC	TET	CAATTACACATGCACATCTCAATTACAC ATGTACATCTG	FAM	CAATTACACATGCACATCT
13_27.04	GCTCAGAGCAAAGGCAAACACT	TGCCACCCACCCAAGTAG	FAM	CCCATCTATGCAGTTC	TET	ATCCCATCTGTGCAGTT
9_21.20	GGAGAAGACTTCCTCCATACAGA	CCCAGCAGGAGAGGCTAA	FAMC	AGGGCGCCAGGCC	TET	ACATACTGGAGGGTGCCAG
10_80.19	GGCAGCAACAATGTGTGAGA	ATGCAGCATCGCTCTGTCTTG	FAM	CAATGCACCCATTAAGTAC	TET	AACAATGCACCCTTTAAGTA
11_04.40	CAACCCTGGTTGCACAAATGAG	CCTTAGGAAGGGCATGGTTCTA	TETC	CAGTGGGTTTGCTTAGTAAGCTCAGTG GGTTTGTTTAGTAA	FAM	CTCAGTGGGTTTGTTTAGTAACAGTGG GTTTGCTTAGTAAG
16_17.32	GGACTCCTACAAACACCCTGA	CTTTGTCCTGTCTGTGCCTTA	FAM	ATGCTCATGTCCCTTAAA	TET	TGCTCATGTTCCTTAAA

Supplementary References

1. Mianné, J. *et al.* Correction of the auditory phenotype in C57BL/6N mice via CRISPR/Cas9-mediated homology directed repair. *Genome Med* **8**, 16 (2016).
2. Hayot, G., Massonot, M., Keime, C., Faure, E. & Golzio, C. Loss of autism-candidate CHD8 perturbs neural crest development and intestinal homeostatic balance. *Life Sci. Alliance* **6**, e202201456 (2023).
3. Lalonde, R., Filali, M. & Strazielle, C. SHIRPA as a Neurological Screening Battery in Mice. *Current Protocols* **1**, (2021).
4. Patange, S. & Maragh, S. Fire Burn and Cauldron Bubble: What Is in Your Genome Editing Brew? *Biochemistry* acs.biochem.2c00431 (2022) doi:10.1021/acs.biochem.2c00431.
5. Sigmon, J. S. *et al.* Content and Performance of the MiniMUGA Genotyping Array: A New Tool To Improve Rigor and Reproducibility in Mouse Research. *Genetics* **216**, 905–930 (2020).
6. Matsuda, K. PCR-Based Detection Methods for Single-Nucleotide Polymorphism or Mutation. in *Advances in Clinical Chemistry* vol. 80 45–72 (Elsevier, 2017).
7. Xu, L. *et al.* Systematic profiling of short tandem repeats in the cattle genome. *Genome Biol Evol* evw256 (2016) doi:10.1093/gbe/evw256.
8. Gurumurthy, C. B. *et al.* Validation of Simple Sequence Length Polymorphism Regions of Commonly Used Mouse Strains for Marker Assisted Speed Congenics Screening. *International Journal of Genomics* **2015**, 1–17 (2015).
9. Berger, B. *et al.* Dog breed affiliation with a forensically validated canine STR set. *Forensic Science International: Genetics* **37**, 126–134 (2018).
10. Pauciullo, A. *et al.* Casein Gene Cluster in Camelids: Comparative Genome Analysis and New Findings on Haplotype Variability and Physical Mapping. *Front. Genet.* **10**, 748 (2019).
11. Manee, M. M. *et al.* Genome-wide characterization and analysis of microsatellite sequences in camelid species. *Mamm Res* **65**, 359–373 (2020).
12. Morgan, A. P. *et al.* The Mouse Universal Genotyping Array: From Substrains to Subspecies. *G3 Genes|Genomes|Genetics* **6**, 263–279 (2016).
13. Ryder, E. *et al.* Genomic analysis of a novel spontaneous albino C57BL/6N mouse strain. *genesis* **51**, 523–528 (2013).
14. Lilue, J. *et al.* Sixteen diverse laboratory mouse reference genomes define strain-specific haplotypes and novel functional loci. *Nat Genet* **50**, 1574–1583 (2018).
15. Kusumi, K. *et al.* Developing a community-based genetic nomenclature for anole lizards. *BMC Genomics* **12**, 554 (2011).
16. Burt, D. W. *et al.* The chicken gene nomenclature committee report. *BMC Genomics* **10**, S5 (2009).
17. Kamau, E. *et al.* Development of a TaqMan Allelic Discrimination Assay for detection of Single Nucleotides Polymorphisms associated with anti-malarial drug resistance. *Malar J* **11**, 23 (2012).
